# Sequence-structure based prediction of pathogenicity for amino acid substitutions in proteins associated with primary immunodeficiencies

**DOI:** 10.3389/fimmu.2025.1492751

**Published:** 2025-02-05

**Authors:** Ekaterina S. Porfireva, Anton D. Zadorozhny, Anastasia V. Rudik, Dmitry A. Filimonov, Alexey A. Lagunin

**Affiliations:** ^1^ Department of Bioinformatics, Pirogov Russian National Research Medical University, Moscow, Russia; ^2^ Laboratory of Structure-Function Based Drug Design, Institute of Biomedical Chemistry, Moscow, Russia

**Keywords:** primary immunodeficiencies, amino acid substitutions, pathogenicity prediction, sequence-structure-property relationships, human genetic variation, SAV-Pred, MultiPASS

## Abstract

**Introduction:**

Primary immunodeficiencies (PIDs) are a group of rare genetic disorders characterized by dysfunction of the immune system components. Early diagnosis and treatment are essential to prevent severe or life-threatening complications. PIDs are manifested by diverse clinical symptoms, posing challenges for accurate diagnosis. A key aspect of PID diagnosis is identifying specific amino acid substitutions in the proteins related with heritable diseases. In this study, we have developed classification sequence-structure-property relationships (SSPR) models for predicting the pathogenicity of amino acid substitutions (AAS) in 25 proteins associated with the most important and genetically studied PIDs and encoded genes: *IL2RG, JAK3, RAG1, RAG2, ADA, DCLRE1C, CD40LG, WAS, ATM, STAT3, KMT2D, BTK, FOXP3, AIRE, FAS, ELANE, ITGB2, CYBB, G6PD, GATA2, STAT1, IFIH1, NLRP3, MEFV, and SERPING1*.

**Methods:**

The data on 4825 pathogenic and benign AASs in the selected proteins were extracted from ClinVar and gnomAD. SSPR models were created for each protein using the MultiPASS software based on the Bayesian algorithm and different levels of MNA (Multilevel Neighborhoods of Atoms) descriptors for the representation of structural formulas of protein fragments including AAS.

**Results:**

The accuracy of prediction was assessed through a 5-fold cross-validation and compared to other bioinformatics tools, such as SIFT4G, Polyphen2 HDIV, FATHMM, MetaSVM, PROVEAN, ClinPred, and Alpha Missense. The best SSPR models demonstrated high accuracy, with an average ROC AUC of 0.831 ± 0.037, a Balanced accuracy of (0.763 ± 0.034), MCC (0.457 ± 0.06), and F-measure (0.623 ± 0.07) across all genes, outperforming the most popular bioinformatics tools.

**Conclusions:**

The best created SSPR models for the prediction of pathogenicity of amino acid substitutions related with PIDs have been implemented in a freely available web application SAV-Pred (Single Amino acid Variants Predictor, http://www.way2drug.com/SAV-Pred/), which may be a useful tool for medical geneticists and clinicians. The use of SAV-Pred for some clinical cases of PIDs are provided.

## 1 Introduction

Primary immunodeficiencies (PIDs), also called inborn errors of immunity (IEI), represent a group of diseases caused by germline mutations. These diseases are caused by mutations in genes encoding proteins that play a crucial role in the functioning of the human immune system. They often lead to a decrease or impairment of the expression of a gene or to its enhancement. The clinical symptoms of PIDs are diverse. Most commonly, they manifest as an increased susceptibility to severe infections. They can also cause autoimmune and autoinflammatory diseases, oncological pathologies, and diseases leading to the development of angioedema. In addition, pathologies related to immune dysregulation and bone marrow failure are included here (1).

Mutations in genes encoding proteins that play a crucial role in the functioning of the human immune system are the reason for PIDs. Early identification of these genetic defects is urgent for successful treatment. Failure to promptly recognize and address these mutations can result in chronic or fatal outcomes. These disorders are often manifested by varied and nonspecific symptoms, leading to significant delays in diagnosis that can last for years. Despite advancements in understanding of these diseases, there continues to be a significant lag in diagnosing primary immunodeficiencies, even in developed countries (2). Timely detection and intervention are essential in managing these conditions and improving patient outcomes. Therefore, it is necessary to accurately identify the pathogenicity of amino acid substitutions to understand the underlying mechanisms of disease.

The advent of Next Generation Sequencing (NGS) technology has revolutionized genetic testing by allowing for the efficient and economical analysis of the entire exome or genome. However, the sheer volume of data produced by NGS can be daunting, particularly when it comes to distinguishing between pathogenic variations and harmless polymorphisms. Unannotated variants or variants of uncertain significance (VUS) create challenges in the diagnosis and treatment of genetic disorders. These variants may not be documented in genetic databases or literature, making it difficult to determine their significance. Bioinformatics analysis methods are essential for resolving VUS, with machine learning-based computational approaches proving to be valuable tools in this process. By analyzing large datasets of biological sequences, researchers can develop predictive models for assessing the pathogenicity of these variants. Despite the availability of numerous pathogenicity predictors like SIFT, Polyphen, and others, no universally accepted algorithm has gained wide recognition in the scientific community (3).

In this study, we present a new version of the SAV-Pred (Single Amino acid Variants Predictor) web application for predicting the pathogenicity of amino acid substitutions in proteins associated with PIDs based on sequence-structure property relationships (SSPR) modeling. This approach was initially used to predict the pathogenic effect of single amino acid substitutions in proteins associated with twenty-five monogenic inherited diseases from the Uniform Screening Panel for Major Conditions recommended by the Advisory Committee on Hereditary Disorders in Newborns and Children (4). The method has now been used for primary immunodeficiencies.

SSPR modeling is focused on machine learning-based classification models that use structural formulas of protein fragments as amino acid sequences description. This methodology allows us to study the way that a protein’s amino acid sequence affects its three-dimensional structure and ultimately determines its properties. It has been successfully applied to predict posttranslational protein phosphorylation sites (5), the association of CDR3 regions of T-cell receptors with MHC epitopes and alleles (6), and amino acid substitutions associated with drug resistance (7).

## 2 Materials and methods

### 2.1 Gene selection

Proteins related to primary immunodeficiencies were chosen based on the IUIS classification (2022) (8) and the prevalence of diseases in the population. The registries selected for the study of immunodeficiency epidemiology were: NAEPID – National Association of Experts in Primary Immunodeficiency in Russia (9); ESID – European Society for Immunodeficiencies registry (10–15); MENA – registry of Middle East and North African countries (16–18); USIDNET – registry of the United States of America (19); PIDJ – Japanese registry of PID patients (20). In addition to these registries, the research on epidemiology PIDs also focused on several countries, such as China (21), South India (22), and Bulgaria (23). Based on the data gathered from these sources, the study identified the most prevalent and socially impactful diseases linked to primary immunodeficiencies, along with the specific genes associated with them. As a result, the most common and socially significant diseases associated with PIDs and their corresponding genes were identified: *IL2RG, JAK3, RAG1, RAG2, ADA, DCLRE1C, CD40LG, WAS, ATM, STAT3, KMT2D, BTK, FOXP3, AIRE, FAS, ELANE, ITGB2, CYBB, G6PD, GATA2, STAT1, IFIH1, NLRP3, MEFV, MVK, SERPING1*.

### 2.2 Preparation of datasets

Data on amino acid substitutions (AASs) in the selected proteins associated with PIDs were obtained using ClinVar (available as of July 2023) (24). Only missense variants were analyzed. Missense mutations are a type of mutation in which one nucleotide in a gene is replaced by another, leading to one AAS in the protein sequence. These AASs can have various consequences for the function of the protein: some missense variants can be pathogenic, leading to impairment of normal protein function, while others can be benign, not leading to diseases. To train classifiers accurately, we decided that each protein should have at least 75 missense SNPs (Single Nucleotide Polymorphism). This threshold is a balance between including as many proteins associated with PIDs as possible and having enough annotated variants to generate accurate and robust models, as well as performing a quality assessment of their accuracy. To supplement the dataset with benign variants, gnomAD (25) was used, with the criterion that the gnomAD allele frequency of the variant was greater than the frequency of the disease in the population (from OMIM database). Datasets containing pathogenic and benign AASs were compiled for all selected proteins.

**Figure 1:**
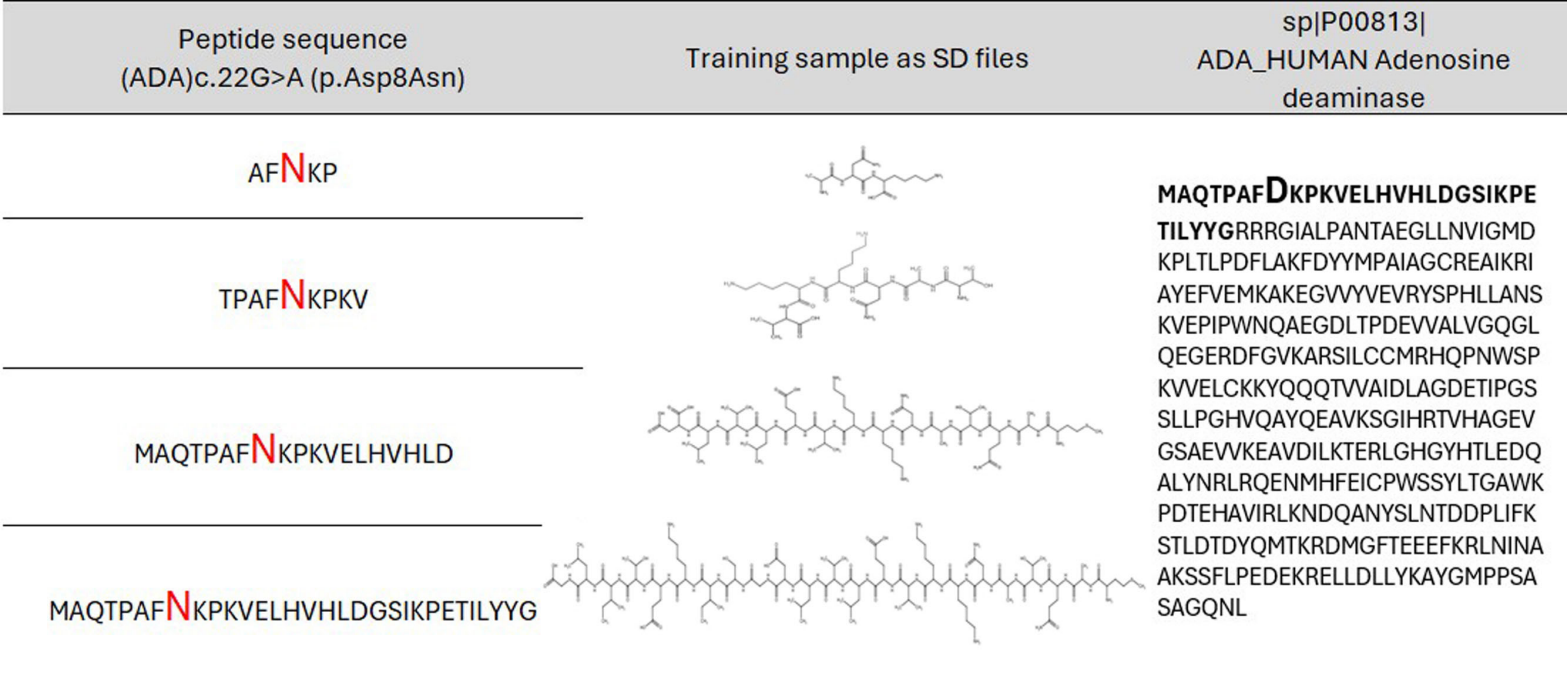
Example of peptides of the appropriate length with amino acid substitution in the center for AAS from ADA protein. Aspartic acid (D) is replaced with asparagine (N).

Based on the collected data, the training sets were created for each protein in the form of MDL SD (structure-data) files with structural formulas of peptides with varying lengths. Machine learning algorithms were trained on these sets. Initially, it was unknown what peptide length would provide the best accuracy for the created classification models of the appropriate protein. Therefore, 14 sets of peptides of different lengths with substitutions in the center, ranging from 5 (two residues in each side from AAS) to 31 (fifteen residues in each side from AAS) amino acid residues with an odd number, were created for each protein (**Figure 1**). If AAS is located at the edge of the sequence, then the number of residues on one side will be less than on the other side for the selected peptide length (for example, two bottom records in **Figure 1**).

### 2.3 SSPR modeling and validation

**Figure 2:**
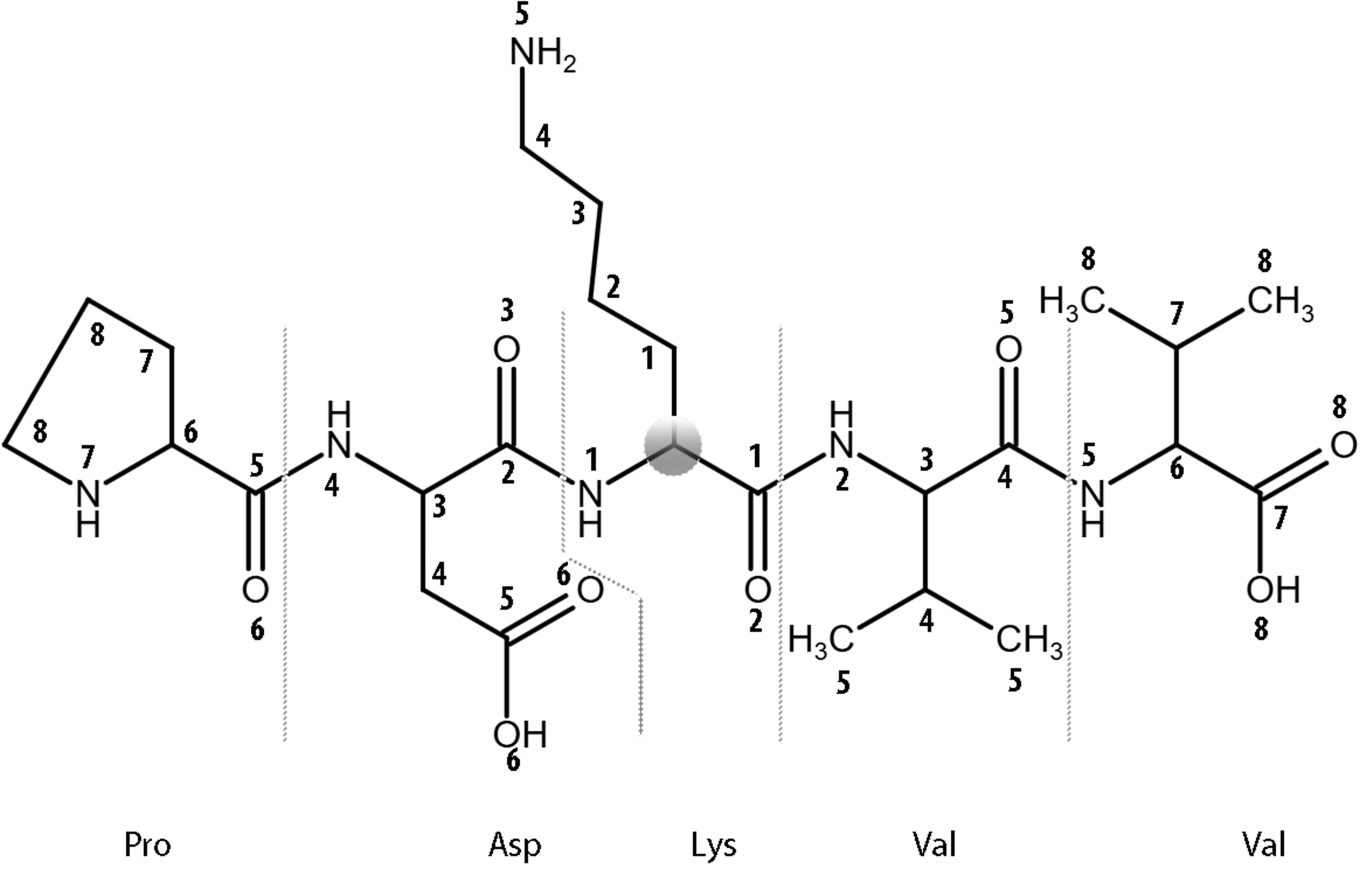
The levels of MNA descriptors beginning from the Cα atom (zero level) of a lysine residue within the central residue of PDKVV peptide from ADA protein are shown.

The proposed approach represents protein molecular fragments with amino acid substitutions as a structural formula and creates the classification “sequence-structure-property relationships” (SSPR) models. Classification models were trained and verified using a modified command line version of the Prediction of Activity Spectra for Substances (PASS) software - MultiPASS (version 2022) (26). It utilizes a standardized set of MNA (Multilevel Neighborhoods of Atoms) descriptors representing the structural formula of peptides and a modified classifier based on the Naive Bayes approach for modeling “structure-property” relationships. This version allows for the utilization of up to 15 levels of MNA descriptors. The MNA descriptor is a way to represent a molecule fragment, including the central atom (except for the hydrogen atom) and the atoms bonded to it. The set of MNA descriptors is used for description of each structural formula. The level of the MNA descriptor reflects the number of covalent bonds from the central atom (**Figure 2**). For constructing SSPR models on 14 distinct peptide fragment length datasets (from 7 to 31 with odd numbers), each of the 11 MNA levels (from 5 to 15) was utilized.

MultiPASS prediction results are a list of predicted characteristics of molecules with Pa (probability of “to be active”) and Pi (probability of “to be inactive”) values. In this study, the Pa value is the probability that the peptide with AAS belongs to the class of pathogenic variants, and the Pi value is the probability that the peptide with AAS does not belong to the class of pathogenic variants. The Pa-Pi value (Confidence) is used for the representation of prediction results in the SAV-Pred web application. Any positive value of Confidence shows that AAS belongs to the class of pathogenic AASs. A higher Confidence value indicates a greater association between AAS and a disease. Any negative value of Confidence means that AAS belongs to the benign class of AASs. The high negative value of Confidence implies the high probability of AAS belonging to benign variants.

The evaluation of model quality for each dataset was conducted using the Invariant Accuracy of Prediction (IAP) implemented in MultiPASS. IAP is an assessment of the probability that positive and negative examples randomly selected from the test set can be correctly classified by a model. Numerically, it is equal to the ROC AUC value traditionally used to assess the accuracy of classification models. The quality of the models for each dataset was evaluated through the leave-one-out (AUC_LOO CV_) and 5-fold cross-validation (AUC_5F CV_) procedures.

## 3 Results

### 3.1 Analysis and selection of SSPR models

The final dataset consisted of 4825 missense variants across 26 proteins associated with primary immunodeficiencies encoded by genes: *IL2RG, JAK3, RAG1, RAG2, ADA, DCLRE1C, CD40LG, WAS, ATM, STAT3, KMT2D, BTK, FOXP3, AIRE, FAS, ELANE, ITGB2, CYBB, G6PD, GATA2, STAT1, IFIH1, NLRP3, MEFV, MVK, and SERPING1* (**Table 1**).

**Table 1:** The list of investigated proteins with associated diseases, data on training sets, and parameters of SSPR models.

Gene	Disease	OMIM	UniProt	P	B	B+	Total	Frequency	PL	MNA	AUC_LOOCV_	AUC_5FCV_
*ADA*	ADA-SCID, Adenosine deaminase deficiency	*102700*	P00813	42	31	41	114	0.0000050	9	5	0.835	0.887
*AIRE*	APECED, Autoimmune polyendocrinopathy syndrome	*240300*	O43918	28	14	90	132	0.0000100	13	6	0.816	0.817
*ATM*	AT, Ataxia-telangiectasia	*208900*	Q13315	66	15	111	192	0.0000050	5	10	0.778	0.768
*BTK*	XLA, Agammaglobulinemia X-linked	*300755*	Q06187	43	9	42	94	0.0000050	29	15	0.813	0.802
*CD40LG*	HIGM1, HYPER-IgM immunodeficiency	*308230*	P29965	38	20	32	90	0.0000050	23	15	0.866	0.870
*CYBB*	CGDX, Chronic granulomatous disease	*306400*	P04839	8	25	81	114	0.0000050	17	6	0.774	0.781
*DCLRE1C*	SCIDA, Severe combined immunodeficiency athabaskan-type, Artemis deficiency	*602450*	Q96SD1	31	10	41	82	0.0000010	31	11	0.846	0.844
*ELANE*	SCN1, Severe congenital neutropenia	*202700*	P08246	37	17	85	139	0.0000040	31	15	0.871	0.887
*FAS*	ALPS-FAS, Autoimmune lymphoproliferative syndrome	*601859*	P25445	30	57	174	261	0.0000020	19	11	0.892	0.842
*FOXP3*	IPEX, Immunodeficiency polyendocrinopathy and enteropathy X-linked	*304790*	Q9BZS1	55	105	2	162	0.0000010	27	7	0.827	0.842
*G6PD*	Anemia, nonspherocytic hemolytic, due to g6pd deficiency	*300908*	P11413	56	671	163	890	0.0000313	13	14	0.711	0.725
*GATA2*	IMD21, immunodeficiency 21, monocytopenia and mycobacterial infection syndrome	*614172*	P23769	87	21	47	155	0.0000053	23	15	0.975	0.978
*IFIH1*	AGS7, aicardi-goutieres syndrome 7	*615846*	Q9BYX4	26	45	29	100	0.0000010	13	6	0.924	0.892
*IL2RG*	SCID, Severe combined immunodeficiency, x-linked	*300400*	P31785	45	18	31	94	0.0000020	17	6	0.834	0.845
*ITGB2*	LAD1, Leukocyte adhesion deficiency	*116920*	P05107	26	7	77	110	0.0000020	21	15	0.756	0.452
*JAK3*	SCID, Severe combined immunodeficiency, autosomal recessive	*600802*	P52333	53	11	88	152	0.0000040	17	10	0.851	0.866
*KMT2D*	Kabuki syndrome	*147920*	O14686	23	19	261	303	0.0000010	31	15	0.942	0.946
*MEFV*	FMF, Familial mediterranean fever	*249100*	O15553	53	34	77	164	0.0000100	23	7	0.833	0.826
*MVK*	HIDS, HYPER-IgD syndrome	*260920*	Q03426	194	13	1	208	0.0000100	19	5	0.652	0.789
*NLRP3*	FCAS, Familial cold autoinflammatory syndrome	*120100*	Q96P20	43	12	297	352	0.0000010	25	13	0.899	0.908
*RAG1*	SCID, Severe combined immunodeficiency, autosomal recessive	*601457*	P15918	82	6	79	167	0.0000010	19	15	0.849	0.768
*RAG2*	SCID, Severe combined immunodeficiency, autosomal recessive	*179616*	P55895	10	60	6	76	0.0000100	31	6	0.790	0.785
*SERPING1*	HAE, Angioedema hereditary	*106100*	P05155	50	13	256	319	0.0000033	25	5	0.812	0.828
*STAT1*	IMD31A, immunodeficiency 31a, autosomal dominant stat1 deficiency	*600555*	P42224	13	17	49	79	0.0000100	23	15	0.891	0.893
*STAT3*	HIES, HYPER-Ig syndrome	*147060*	P40763	65	7	71	143	0.0000050	21	15	0.894	0.884
*WAS*	WAS, Wiskott-aldrich syndrome	*301000*	P42768	46	23	64	133	0.0000050	31	15	0.865	0.868
Mean											0.838	0.831

B, Benign variants in the sets; P, Pathogenic variants in the sets; B+, benign variants that initially did not have clinical classification and added from gnomAD; AUC_LOOCV_, AUC obtained by leave-one out validation procedure; AUC_5FCV_, AUC obtained by 5-fold cross-validation procedure; Frequency, frequency of the disease in the population (OMIM); PL, peptide length; MNA (the level of MNA descriptors), parameters of sequence–structure–property relationships (SSPR) models.

**Figure 3:**
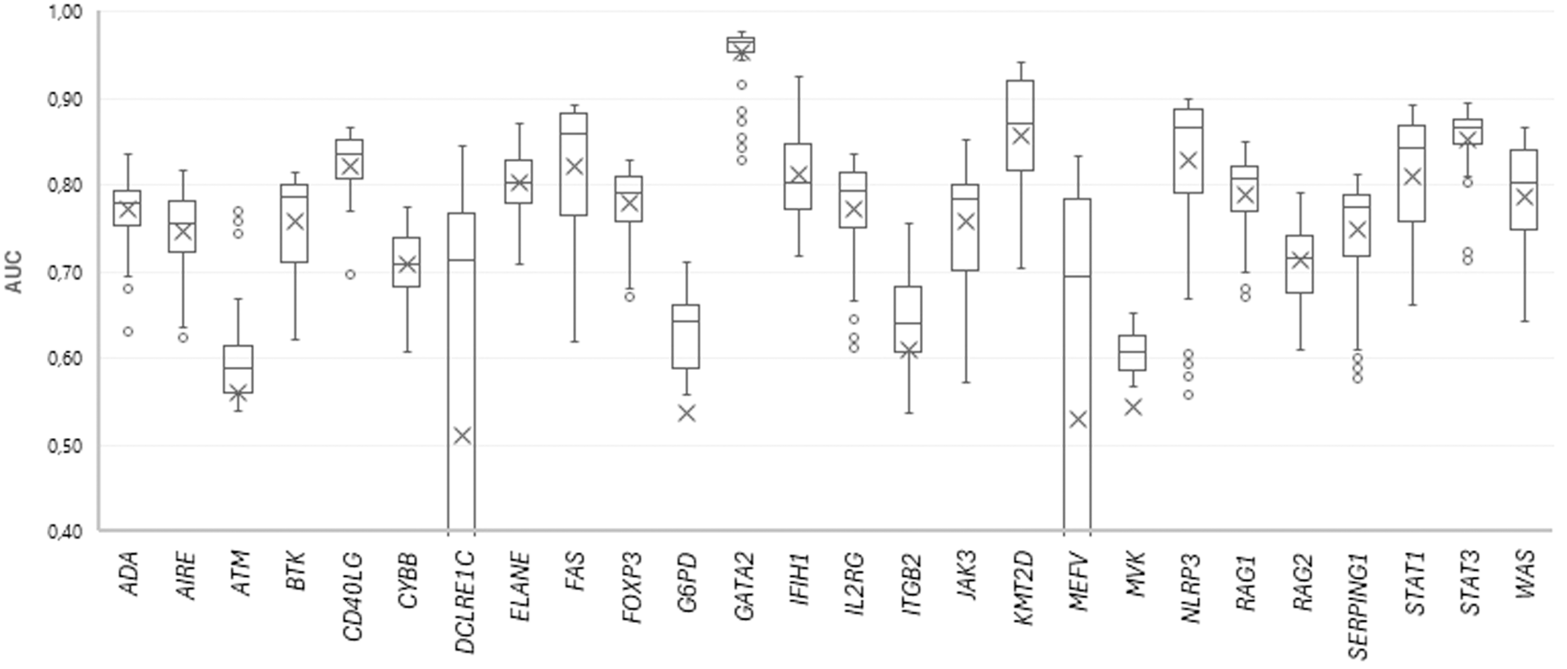
The plot of AUC values for SSPR protein models at different MNA descriptor levels and peptide lengths.

154 SSPR models with different levels of MNA descriptors (11 levels, from 5 to 15) and different peptide lengths (14 length values with odd numbers, from 5 to 31 amino acid residues) were developed for each protein. The quality of SSPR models for each dataset was evaluated through the leave-one-out cross-validation procedure (AUC_LOO CV_) (**Figure 3**).

**Figure 4:**
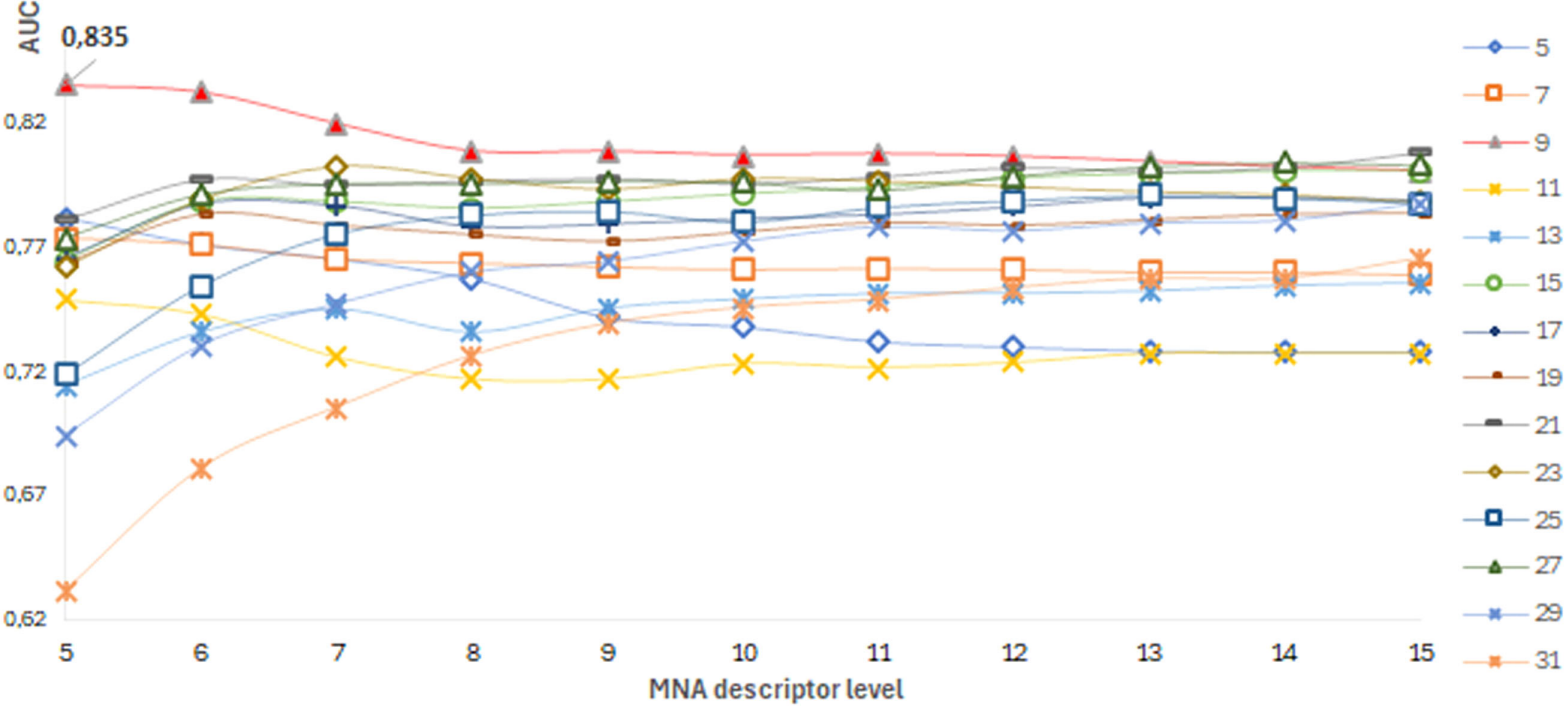
ADA protein. A dependence of the SSPR model’s accuracy on the level of MNA descriptors and different peptide length values.

The best SSPR model for predicting pathogenicity for each protein was selected based on the highest AUC_LOO CV_ values of models built on different training datasets of amino acid substitutions. The distribution of AUC_LOO CV_ values of the SSPR models created on different peptide length and levels of MNA descriptors for ADA protein are shown as an example (**Figure 4**).

Among the best SSPR models, the average AUC_LOO CV_ value was 0.838 ± 0.027. **Figure 3** shows that the best SSPR models exceeded 0.80 for 20 proteins. The high accuracy levels (above 0.920) of the SSPR models for genes *IFIH1, KMT2D*, and *GATA2* indicate their potential effectiveness in prediction. However, the SSPR model for the MVK protein showed a value below 0.7 (0.652). It may be related with a disbalance of data (Pathogenic variants – 194 and Benign variants – 14) in the training set (**Table 1**). The best SSPR models were selected for implementation in the SAV-Pred web application (hereinafter, we will call our method SAV-Pred). These models additionally underwent a thorough evaluation using a 5-fold cross-validation (5F CV) procedure. This rigorous testing process ensured that the algorithm was robust and reliable in predicting the outcomes’ validity. The average AUC_5F CV_ score was 0.831 ± 0.037, which indicates a high level of prediction accuracy (**Table 1**).

### 3.2 Comparison the best SSPR models with other bioinformatics tools

The prediction accuracy obtained through the 5-fold cross-validation was compared to the accuracy of other well-known bioinformatics tools (SIFT4G, Polyphen2_HDIV, FATHMM, AlphaMissense, ClinPred, MetaSVM, and PROVEAN) for the same variations, based on data from dbNSFP4 (27–33). The numerical values for the predictive indicators in **Tables 2**–**5** were derived from dbNSFP4. Data processing and the calculation of model performance metrics were made using the KNIME software. SAV-Pred (0.831 ± 0.037) was among the top three in terms of AUC values, along with ClinPred (0.846 ± 0.050) and AlphaMissense (0.839 ± 0.047). However, SAV-Pred has shown the best prediction results for 10 proteins encoded by the genes *JAK3, ATM, KMT2D, FOXP3, AIRE, ELANE, STAT1, IFIH1, NLRP3, and MEFV*, while ClinPred and Alpha Missense were better for 8 (*CD40LG, CYBB, FAS, IL2RG, ITGB2, SERPING1, STAT3, WAS*) and 4 (*ADA, BTK, RAG1, RAG2*), proteins (**Table 2**).

**Table 2:** Accuracy comparison of the tools in predicting single amino acid substitution effects in proteins related to PIDs on the AUC metric.

Gene	SAV-Pred	SIFT4G	Polyphen2	FATHMM	MetaSVM	PROVEAN	ClinPred	Alpha Missense
*ADA*	0.887	0.862	0.890	0.644	0.888	0.791	0.791	**0.907**
*AIRE*	**0.817**	0.796	0.656	0.628	0.758	0.599	0.740	0.781
*ATM*	**0.768**	0.685	0.522	0.407	0.403	0.596	0.667	0.576
*BTK*	0.802	0.929	0.841	0.758	0.934	0.903	0.959	**0.960**
*CD40LG*	0.870	0.772	0.908	0.888	0.942	0.914	**0.980**	0.971
*CYBB*	0.781	0.876	0.844	0.741	0.846	0.895	**0.964**	0.959
*DCLRE1C*	0.844	0.814	0.734	0.825	**0.958**	0.888	0.954	0.934
*ELANE*	**0.887**	0.782	0.745	0.765	0.814	0.767	0.816	0.839
*FAS*	0.842	0.821	0.777	0.622	0.807	0.823	**0.888**	0.870
*FOXP3*	**0.842**	0.746	0.705	0.409	0.764	0.708	0.768	0.763
*G6PD*	0.725	0.702	0.752	0.746	0.644	**0.784**	0.661	0.669
*GATA2*	0.978	0.966	0.888	**0.999**	0.782	0.898	0.997	0.995
*IFIH1*	**0.892**	0.613	0.709	0.609	0.718	0.694	0.880	0.796
*IL2RG*	0.845	0.770	0.917	0.516	0.800	0.846	**0.973**	0.923
*ITGB2*	0.452	0.833	–	0.820	0.894	0.879	**0.899**	0.888
*JAK3*	**0.866**	0.746	0.775	0.509	0.727	0.695	0.798	0.790
*KMT2D*	**0.946**	–	0.862	0.789	0.861	0.892	0.941	0.878
*MEFV*	**0.826**	0.317	0.372	0.582	0.539	0.292	0.453	0.479
*MVK*	0.789	0.783	0.774	0.623	**0.818**	0.721	0.757	0.765
*NLRP3*	**0.908**	0.605	0.526	0.602	0.705	0.520	0.719	0.735
*RAG1*	0.768	0.820	0.792	0.647	0.736	0.754	0.827	**0.861**
*RAG2*	0.785	0.860	0.769	0.614	0.797	0.860	0.862	**0.891**
*SERPING1*	0.828	0.871	0.827	0.623	0.919	0.858	**0.985**	0.958
*STAT1*	**0.893**	0.732	0.751	0.470	0.652	0.764	0.837	0.847
*STAT3*	0.884	0.836	0.758	0.777	0.853	0.822	**0.928**	0.855
*WAS*	0.868	0.874	0.919	0.514	0.641	0.872	**0.951**	0.924
**Mean**	0.831	0.746	0.731	0.659	0.777	0.771	**0.846**	0.839

For each gene, the best results are highlighted in bold.

**Table 3:** The accuracy comparison of the tools in predicting single amino acid substitution effects in proteins related to PIDs on the BA metric.

Gene	SAV-Pred	SIFT4G	Polyphen2	FATHMM	MetaSVM	PROVEAN	ClinPred	Alpha Missense
*ADA*	0.793	0.814	0.783	0.500	0.615	0.729	0.729	**0.850**
*AIRE*	**0.745**	0.734	0.604	0.567	0.655	0.637	0.669	0.723
*ATM*	**0.645**	0.572	0.468	0.487	0.491	0.523	0.563	0.568
*BTK*	0.735	**0.853**	0.751	0.613	0.759	0.820	0.770	0.791
*CD40LG*	0.769	0.741	0.763	0.691	0.776	0.827	0.863	**0.916**
*CYBB*	0.686	0.756	0.768	0.500	0.654	0.780	0.817	**0.822**
*DCLRE1C*	0.887	0.729	0.705	0.574	0.825	**0.889**	0.808	0.827
*ELANE*	**0.823**	0.731	0.695	0.500	0.792	0.660	0.720	0.729
*FAS*	0.772	0.731	0.735	0.540	0.797	0.745	**0.834**	0.808
*FOXP3*	**0.795**	0.613	0.627	0.500	0.615	0.689	0.721	0.648
*G6PD*	**0.749**	0.581	0.684	0.500	0.494	0.701	0.649	0.575
*GATA2*	**0.930**	0.868	0.684	0.500	0.534	0.856	0.575	0.799
*IFIH1*	**0.809**	0.616	0.679	0.500	0.638	0.692	0.776	0.638
*IL2RG*	0.763	0.661	0.756	0.469	0.606	0.803	**0.869**	0.817
*ITGB2*	0.460	**0.786**	–	0.584	0.728	0.776	0.748	0.747
*JAK3*	**0.782**	0.702	0.676	0.451	0.673	0.667	0.725	0.695
*KMT2D*	0.852	–	0.786	0.756	0.811	**0.862**	0.850	0.819
*MEFV*	**0.725**	0.446	0.354	0.485	0.500	0.362	0.454	0.508
*MVK*	0.729	0.729	0.706	0.500	**0.731**	0.726	0.692	0.724
*NLRP3*	**0.830**	0.558	0.471	0.556	0.613	0.506	0.588	0.602
*RAG1*	0.697	0.672	0.699	0.555	0.730	0.662	0.678	**0.783**
*RAG2*	0.721	0.686	0.725	0.622	0.696	0.730	0.668	**0.801**
*SERPING1*	0.714	0.799	0.709	0.500	0.873	0.840	0.877	**0.918**
*STAT1*	**0.791**	0.725	0.754	0.432	0.612	0.680	0.747	0.766
*STAT3*	**0.831**	0.776	0.655	0.654	0.748	0.738	0.698	0.731
*WAS*	0.806	0.740	0.794	0.521	0.543	0.821	0.784	**0.858**
**Mean**	**0.763**	0.678	0.655	0.541	0.674	0.720	0.726	0.748

For each gene, the best results are highlighted in bold.

**Table 4:** The accuracy comparison of the tools in predicting single amino acid substitution effects in proteins related to PIDs on the MCC metric.

Gene	SAV-Pred	SIFT4G	Polyphen2	FATHMM	MetaSVM	PROVEAN	ClinPred	Alpha Missense
*ADA*	0.578	0.613	0.569	0.000	0.323	0.471	0.471	**0.681**
*AIRE*	**0.490**	0.466	0.218	0.206	0.320	0.274	0.337	0.462
*ATM*	**0.164**	0.086	-0.039	-0.049	-0.018	0.033	0.073	0.081
*BTK*	0.468	**0.742**	0.571	0.238	0.584	0.664	0.630	0.635
*CD40LG*	0.527	0.468	0.526	0.379	0.539	0.649	0.699	**0.816**
*CYBB*	0.353	0.487	0.523	0.000	0.353	0.533	0.608	**0.611**
*DCLRE1C*	**0.440**	0.212	0.187	0.206	0.358	0.401	0.283	0.306
*ELANE*	**0.619**	0.438	0.381	0.000	0.553	0.306	0.427	0.436
*FAS*	0.482	0.417	0.424	0.124	0.550	0.454	0.613	**0.630**
*FOXP3*	**0.526**	0.200	0.230	0.000	0.274	0.353	0.397	0.280
*G6PD*	**0.244**	0.078	0.198	0.000	-0.024	0.233	0.176	0.084
*GATA2*	**0.738**	0.243	0.114	0.000	0.041	0.230	0.063	0.180
*IFIH1*	**0.475**	0.141	0.229	0.000	0.428	0.254	0.365	0.197
*IL2RG*	0.512	0.346	0.480	-0.094	0.217	0.566	**0.672**	0.611
*ITGB2*	0.039	**0.286**	–	0.113	0.225	0.272	0.244	0.250
*JAK3*	**0.487**	0.327	0.278	-0.089	0.309	0.268	0.355	0.331
*KMT2D*	**0.464**	–	0.267	0.270	0.364	0.417	0.388	0.360
*MEFV*	**0.359**	-0.080	-0.218	-0.073	0.000	-0.224	-0.071	0.014
*MVK*	**0.463**	0.404	0.380	0.000	0.431	0.403	0.360	0.401
*NLRP3*	**0.521**	0.053	-0.027	0.051	0.119	0.005	0.080	0.104
*RAG1*	0.374	0.378	0.409	0.103	0.445	0.309	0.363	**0.537**
*RAG2*	0.443	0.455	0.467	0.311	0.470	0.462	0.379	**0.603**
*SERPING1*	0.407	0.529	0.371	0.000	0.675	0.619	0.671	**0.830**
*STAT1*	**0.582**	0.435	0.488	-0.132	0.224	0.346	0.493	0.511
*STAT3*	**0.641**	0.455	0.252	0.263	0.399	0.398	0.342	0.373
*WAS*	0.555	0.430	0.525	0.109	0.157	0.571	0.508	**0.661**
**Mean**	**0.457**	0.331	0.300	0.074	0.320	0.356	0.382	0.423

For each gene, the best results are highlighted in bold.

**Table 5:** The accuracy comparison of the tools in predicting single amino acid substitution effects in proteins related to PIDs on the F-measure metric.

Gene	SAV-Pred	SIFT4G	Polyphen2	FATHMM	MetaSVM	PROVEAN	ClinPred	Alpha Missense
*ADA*	0.765	0.773	0.744	0.559	0.623	0.697	0.697	**0.805**
*AIRE*	**0.739**	0.725	0.635	0.656	0.673	0.630	0.659	0.667
*ATM*	**0.229**	0.200	0.127	0.000	0.080	0.154	0.192	0.178
*BTK*	0.757	**0.893**	0.830	0.701	0.834	0.862	0.848	0.852
*CD40LG*	0.716	0.685	0.709	0.641	0.720	0.780	0.812	**0.885**
*CYBB*	0.585	0.682	0.694	0.512	0.601	0.706	**0.743**	0.741
*DCLRE1C*	**0.400**	0.200	0.182	0.222	0.333	0.343	0.231	0.250
*ELANE*	**0.760**	0.651	0.620	0.505	0.715	0.582	0.643	0.642
*FAS*	0.615	0.603	0.605	0.460	0.688	0.625	0.727	**0.731**
*FOXP3*	**0.657**	0.462	0.487	0.425	0.490	0.545	0.581	0.491
*G6PD*	0.831	0.822	0.885	**0.974**	0.968	0.906	0.906	0.883
*GATA2*	**0.762**	0.148	0.068	0.044	0.047	0.138	0.051	0.103
*IFIH1*	**0.533**	0.241	0.306	0.000	0.419	0.326	0.406	0.306
*IL2RG*	0.703	0.541	0.621	0.413	0.496	0.699	**0.761**	0.727
*ITGB2*	0.088	**0.257**	–	0.142	0.209	0.236	0.224	0.244
*JAK3*	**0.603**	0.472	0.430	0.145	0.456	0.432	0.482	0.476
*KMT2D*	**0.460**	–	0.231	0.268	0.355	0.388	0.357	0.343
*MEFV*	**0.474**	0.200	0.120	0.000	0.105	0.054	0.186	0.200
*MVK*	**0.730**	0.579	0.552	0.419	0.574	0.574	0.540	0.580
*NLRP3*	**0.585**	0.122	0.088	0.121	0.167	0.098	0.133	0.156
*RAG1*	0.616	0.586	0.606	0.437	0.632	0.550	0.588	**0.683**
*RAG2*	0.698	0.714	0.727	0.667	0.721	0.692	0.692	**0.787**
*SERPING1*	0.636	0.653	0.562	0.423	0.760	0.724	0.748	**0.876**
*STAT1*	**0.793**	0.654	0.690	0.311	0.505	0.604	0.687	0.703
*STAT3*	**0.774**	0.567	0.427	0.424	0.519	0.531	0.455	0.494
*WAS*	0.681	0.602	0.648	0.438	0.449	0.680	0.636	**0.756**
**Mean**	**0.623**	0.521	0.504	0.431	0.505	0.521	0.538	0.560

For each gene, the best results are highlighted in bold.

SAV-Pred has demonstrated the best performance on metrics used in conditions of imbalanced data. The Balanced Accuracy (BA) metric helps to obtain a more accurate assessment of the model’s quality in conditions of imbalanced data when the number of events in one class significantly exceeds the number of events in another (**Table 3**). Average Balanced (BA) accuracy for SAV-Pred was higher in comparison with other methods and achieved 0.763 ± 0.034. BA of SAV-Pred was the highest for 12 proteins. ClinPred and Alpha Missense were better for 2 and 7 proteins, respectively.

MCC (Matthews correlation coefficient) is a measure of the quality of a model that balances a True Positives Rate and a True Negatives Rate. It is commonly used in cases where it is crucial to balance the prediction performance on both positive and negative samples. The average MCC for our models (SAV-Pred) was also better and achieved 0.457 ± 0.06 (**Table 4**). MCC for SAV-Pred models was the highest for 15 proteins. ClinPred and Alpha Missense were better for 1 and 8 proteins, respectively.

The F-measure is useful when achieving a balance between precision and recall is important, as well as in tasks with imbalanced classes. It is also widely used as a metric for evaluating the quality of machine-learning models related to classification tasks. The average F-measure for SAV-Pred models was also better and achieved 0.623 ± 0.07, confirming the effectiveness and accuracy of this algorithm compared to other predictors (**Table 5**). The F-measure for SAV-Pred models was the highest for 14 proteins. ClinPred and Alpha Missense were better for 2 and 7 proteins, respectively.

The results of the evaluation highlighted the effectiveness and efficiency of the SAV-Pred algorithm in making accurate predictions in the proteins associated with PIDs. These data demonstrate that this method outperformed other tools in terms of prediction accuracy and specificity, highlighting its potential utility in clinical and research settings.

### 3.3 SAV-Pred web application

The best SSPR models for 25 proteins associated with PIDs (the model for *MVK* was excluded) became the basis for the section “Inborn Errors of Immunity” in the SAV-Pred web application predicting the pathogenic effects of amino acid substitutions (AAS) on the way2drug.com portal (4): (http://www.way2drug.com/SAV-Pred/) (accessed on August 30, 2024). Users should press the “Input” button on the Home page and select “One request” or “*.csv file” commands to form a query for prediction of the pathogenicity of amino acid substitution(s). After selecting “One request” in the appeared “Input data” window, users should select “Inborn Errors of Immunity” in the field “Dataset” and then select the gene name (field “Gene”) or disease (field “Disease”) to select the protein where amino acid substitution would be estimated. Then a position and amino acid substitution should be defined for the creation of a query. The “Input data” window also includes information on the maximal position in a sequence of the appropriate protein. These steps may be repeated several times to form a query for the prediction of the pathogenicity for several amino acid substitutions. The selection of “*.csv file” command allows loading a query list of substitutions in the following format:

<gene name> <position> <a.a. substitution>

**Figure 5:**
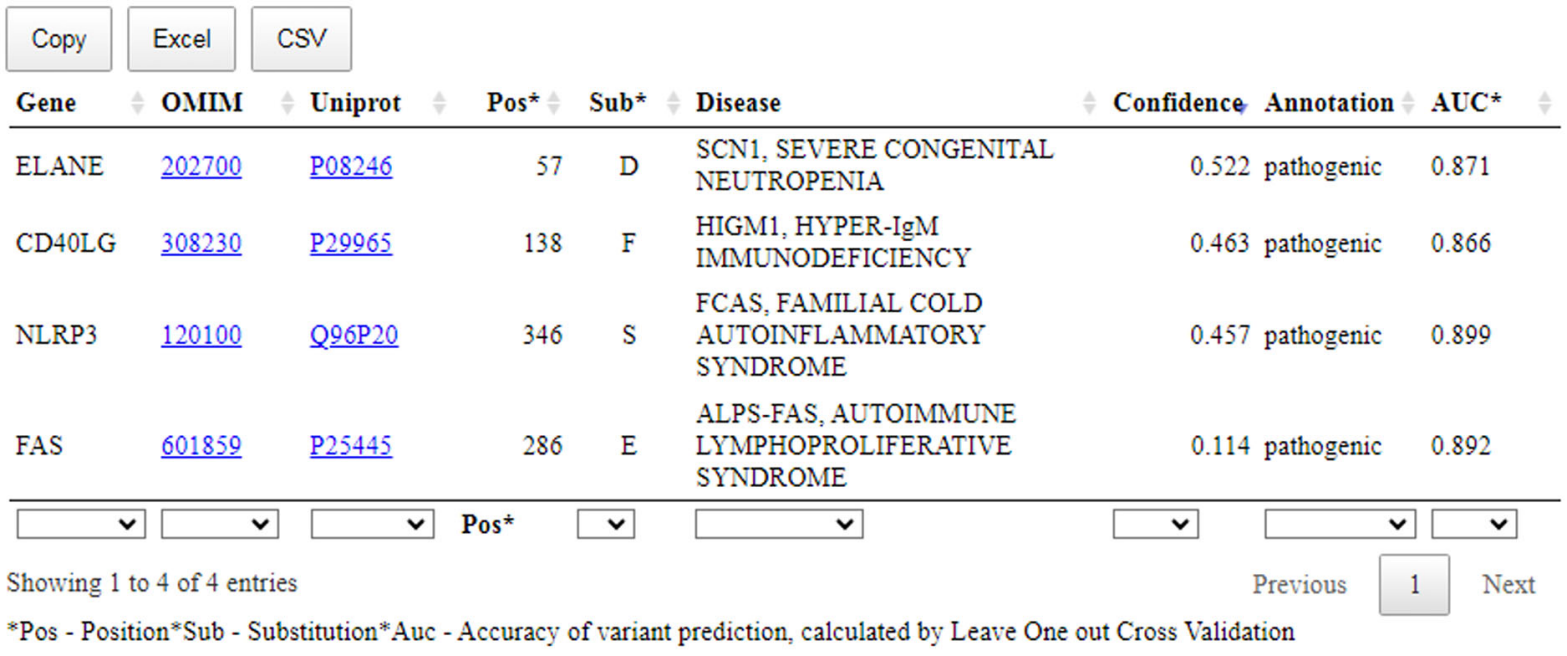
The prediction results of the SAV-Pred for missense variants related with the clinical cases. Pos, position of AAS; Sub, amino acid substitution; Confidence, Pa-Pi value; AUC, the accuracy of prediction of SSPR model (AUC) calculated by leave-one-out cross-validation procedure.

After forming the query, pressing the button “Make prediction” starts the prediction. The prediction results are shown in a table (**Figure 5**). They can be saved as a file in the CSV or XLS formats.

**Figure 5** shows that a table with the prediction results includes: name of the gene; OMIM ID with the hyperlink to the records related with the appropriate disease; UniProt ID with the hyperlink to the records related with the appropriate protein; position with AAS; one-letter code of AAS; Confidence value and AUC value of SSPR model calculated by leave-one-out cross-validation procedure for the appropriate protein.

### 3.4 Clinical cases and SAV-Pred prediction

In modern medicine, predicting the functional consequences of gene variants is becoming increasingly important, especially in the context of classifying variants of uncertain significance (VUS). The SAV-Pred prediction is a promising tool for this. Several clinical cases with VUS from scientific publications have been considered as examples for demonstration of practical usefulness of SAV-Pred for revealing possible pathogenic AASs.

#### 3.4.1 Clinical case 1

Gu and co-authors published the case describing a patient with a *de novo* variant in the *FAS* gene presenting a severe phenotype of ALPS (Autoimmune Lymphoproliferative Syndrome) (34).

*Phenotype*: a 2-year-old boy with clinical characteristics of ALPS, including splenomegaly and lymphadenopathy, and elevated double-negative T-cells (DNT) (6.8%). In healthy individuals, the levels of such cells are not more than 1%. The patient had no family history of the condition.

*Genotype*: a novel heterozygous missense variant in the *FAS* gene was identified (NM_000043.6:c.857G>A, p.G286E, p.Gly286Glu) (34).

In summary, the available evidence is currently insufficient to determine the role of this variant in disease. Therefore, it has been classified as a Variant of Uncertain Significance. This variant was absent in population databases. Aggregated prediction agrees on the potential pathogenic impact of this missense change, SAV-Pred prediction identified this variant as pathogenic (Confidence value: 0.114) (**Figure 5**). Indirect confirmation of the connection of this variant with pathogenicity can be its positive response to treatment with sirolimus (34). Sirolimus is effective in ALPS treatment at pathogenic mutations in FAS.

#### 3.4.2 Clinical case 2

Park and co-authors published the case describing a patient with the variant in the *ELANE* gene presenting episodes of cyclic neutropenia (35).

*Phenotype:* a 20-year-old male with febrile neutropenia. He has a history of cyclic neutropenia since the age of 7, experiencing self-limiting fever attacks with pain and swelling in the buccal region. Blood analysis revealed a leukocyte count of 1.98×10^9^/L (normal lower boundary 4.0×10^9^) and an absolute neutrophil count of 0.26×10^9^/L (normal lower boundary 0.3×10^9^).

*Genotype:* a novel heterozygous missense variant in the *ELANE* gene was identified (NM_001972.4:c.170C>A, p.A57D, p.Ala57Asp) (35).

This variant was not present in ClinVar. The variant can be classified as Likely Pathogenic. This variant has not been reported in population databases. According to Pfam, the variant is located in a critical functional Trypsin domain. *ELANE* gene has low rate of benign missense mutations and for missense variants are a common mechanism of a disease. The known pathogenic variant c.170C>T (p.Ala57Val) has been reported. In-silico predictions are either unavailable or do not agree on the potential impact of this missense change. The SAV-Pred prediction identified this variant as pathogenic (Confidence value: 0.522) (**Figure 5**).

#### 3.4.3 Clinical case 3

Lien and co-authors published the case describing a patient with the variant in the *CD40LG* gene, X-linked hyper IgM syndrome (36).

*Phenotype:* a one-year-old boy with hyper IgM syndrome. Early symptoms included fever, recurrent pneumonia, sepsis, diarrhea, enteritis, and ulcerative colitis. Serum immunoglobulin levels showed decreased IgA (0.32 g/L, normal range 0.4-2.0 g/L) and IgG (3.50 g/L, normal range 4.9-16.1 g/L) levels, but IgM level was high - 3.35 g/L (normal range 0.5-2.0 g/L).

*Genotype*: a new mutation (NM_000074.3:c.414A>T, p.L138F, p.Leu138Phe) was found in the *CD40LG* gene (36).

This variant was not present in ClinVar. The variant can be classified as Likely Pathogenic. This variant has not been reported in population databases. Different amino acid change Leu138Ser as a known pathogenic variant has been reported. The variant is located in exonic mutational hotspot (12 pathogenic or likely pathogenic reported variants were found in a 68bp region surrounding this variant in exon 5 within the region X:136659038-136659106 (GRCh38) without any missense benign variants). *In-silico* predictions are either unavailable or do not agree on the potential impact of this missense change. The SAV-Pred prediction identified this variant as pathogenic (Confidence value: 0.463) (**Figure 5**).

#### 3.4.4 Clinical case 4

Padula and co-authors published the case describing a patient with Behcet’s disease with the variant in the *NLRP3* gene (37).

*Phenotype:* A 62-year-old man with fever and multiple ulcers in the oral cavity. He was also diagnosed with folliculitis, nodular erythema, and arthritis in the lower limbs.

*Genotype*: a new heterozygous missense variant in *NLRP3* gene (NM_001243133.2:c.1037T>G, p.I348S, p.Ile346Ser) was identified (37).

This variant was not present in ClinVar and can be classified as a Variant of Uncertain Significance. The variant has extremely low frequency in gnomAD population databases and computational prediction tools unanimously support a deleterious effect on the gene. The SAV-Pred prediction identified this variant as pathogenic (Confidence value: 0.457) (**Figure 5**).

Thus, SAV-Pred demonstrated remarkable accuracy in classifying clinical cases with suspected pathogenic amino acid substitutions. It may be a valuable tool for both clinicians and geneticists. It holds significant promise for its integration into routine clinical practice and underscores the significance of using advanced computational tools in healthcare to enhance patient outcomes and simplify the diagnostic process.

## 4 Discussion

The use of bioinformatics and machine learning methods is becoming increasingly important for interpreting unannotated variants in genetic studies of rare hereditary diseases. With a large amount of biological sequence data that can be used as training material, these approaches enable the creation of models capable of detecting pathogenic variants even in the absence of annotations. This integration of bioinformatics and machine learning methods offers new opportunities for identifying genetic variants associated with rare diseases and advancing precision medicine.

The SAV-Pred web application represents a unique approach based on the development of machine learning and molecular prediction models. While this approach has previously been employed successfully in predicting the pathogenicity of amino acid substitutions in proteins related to congenital diseases investigated in newborn screening, its application to assessing the pathogenicity of amino acid substitutions in proteins associated with PIDs marks a significant milestone.

Traditionally, the clinical significance of new mutations in genes associated with PIDs could only be presumed based on some publications concerning a particular protein and its mediating gene and universal bioinformatics tools like SIFT or Polyphen. However, the development of a novel tool presented in this study now enables to make the estimation of AASs specifically for PID-associated proteins. A freely available web application empowers geneticists and clinicians to make informed decisions regarding treatment strategies by assessing the pathogenicity of specific amino acid substitutions. Furthermore, the utility of this web application extends beyond clinical settings, offering valuable insights for prospective parents planning pregnancy based on genetic analysis. Informing patients about their genetic risks encourages their active participation in making decisions about their health. Identifying pathogenic genetic variants will help clinicians understand a patient’s condition better. Thus, by predicting potential risks associated with certain genetic variations, this tool can be used to provide personalized recommendations to reduce potential health problems.

Our study also demonstrated that there is no best universal bioinformatic tool for predicting the pathogenicity of AASs. Here, one may see what bioinformatic tool is the best for the estimation of AAS pathogenicity in the appropriate proteins related with PIDs. For more than half of the proteins (14 from 25 proteins), SAV-Pred models showed the best average characteristics of accuracy calculated by the data from **Tables 2**-**5** (*AIRE, ATM, DCLRE1C, ELANE, FOXP3, GATA2, IFIH1, JAK3, KMT2D, MEFV, MVK, NLRP3, STAT1, and STAT3*). At the same time, the advantage in accuracy is absolutely overwhelming for the genes *GATA2, MEFV*, and *NLRP3*, which relate with IMD21 (immunodeficiency 21, monocytopenia and mycobacterial infection syndrome), Familial mediterranean fever, and Familial cold autoinflammatory syndrome, respectively. The average characteristics of accuracy for Alpha Missense were better for 7 proteins related with *ADA, CD40LG, CYBB, RAG1, RAG2, SERPING1*, and *WAS* genes. ClinPred was the best for *FAS* and *IL2RG*, and it revealed comparative accuracy with Alpha Missense for *CYBB*. SIFT4G was the best for *BTK*, and it revealed comparative accuracy with PROVEAN for *ITGB2.* SIFT4G was the best for *G6PD*. Based on the obtained results, we recommend selecting the most accurate models for the corresponding genes and adding SAV-Pred model prediction to the set of prediction programs already used for analyzing potential pathogenic variants. We believe that integrating our predictor with existing tools can enhance the efficiency and accuracy of variant interpretation in clinical settings.

In this study we made models for well-studied mutated genes related with PIDs. There are also many genes associated with PIDs that are poorly characterized in terms of pathogenic and benign mutations. The small data on annotated genetic variants is a limitation for the proposed approach because of the training set is a key factor for successful machine learning models. Our experience displayed that small data on pathogenic and benign AASs in such genes makes the model training less effective and robust. Despite this, annotated data of genetic variants increases over time. Therefore, it is hoped that it will be possible to create good models for predicting pathogenic AASs and for genes associated with PIDs, which currently have a small number of annotated AASs. Looking ahead, SAV-Pred can be constantly improved by adding new genes associated with PIDs. By updating the gene database regularly and expanding its associations with diverse forms of immunodeficiencies, this web application is poised to deliver increasingly accurate and pertinent information, empowering users to make well-informed medical decisions.

## Data Availability

Publicly available datasets were analyzed in this study. The data used for the creation and validation of SAR models were extracted from freely available databases ClinVar (https://www.ncbi.nlm.nih.gov/clinvar/), gnomAD (https://gnomad.broadinstitute.org/), and dbNSFP4 (http://database.liulab.science/dbNSFP). SAV-Pred is a freely available web application available at http://way2drug.com/SAV-pred/.
